# 

**Published:** 1909-01-23

**Authors:** 


### GRADUATES' MEDICAL SCHOOLS.

DIARY FOR WEEK JAN. 25 to JAN. 30.

### HOSPITAL FOR CONSUMPTION AND DISEASES OF THE CHEST, Brompton, S.W.

**Jan. 27,** Dr. Habershon, **Cases from the Wards.**

### ST. JOHN'S HOSPITAL FOR DISEASES OF THE SKIN, Leicester Square, W.C.

**Jan. 28,** Chesterfield Lectures, **Syphilis—I.**

### THE POST-GRADUATE COLLEGE, West London Hospital, Hammersmith, W.

**At 10 a.m.**

**Jan. 25 and 28,** Surgical Registrar, **Demonstration.**

**Jan. 29,** Medical Registrar, **Demonstration.**

**At 12 noon.**

**Jan. 25,** Dr. Low, **Pathological Demonstration.**

**At 12.15 p.m.**

**Jan. 27 and 30,** Dr. Pritchard, **Practical Medicine.**

**At 5 p.m.**

**Jan. 25,** Dr. Davis, **Diseases of Throat, Nose, and Ear.**

**Jan. 26,** Dr. Low, **Ankylostomiasis.**

**Jan. 27,** Dr. Beddard, **Medicine—I.**

**Jan. 28,** Mr. Lloyd, **Anæsthetics—I.**

**Jan. 29,** Dr. Abraham, **Cases of Skin Disease.**

### NATIONAL HOSPITAL FOR THE PARALYSED AND EPILEPTIC, Queen Sq., Bloomsbury, W.C.

**At 3.30 p.m.**

**Jan. 26,** Dr. Grainger Stewart, **The Significance of Focal Fits.**

**Jan. 29,** Dr. Tooth, **Clinical Lecture.**

### THE THROAT HOSPITAL, Golden Square, W.

**At 5.30 p.m.**

**Jan. 25,** Dr. Powell, **Post Nasal Catarrh, Adenoids, and Tumours.**

**Jan. 28,** Dr. Barton, **General Anæsthesia in Operations on the Upper Air Passages.**

### NORTH-EAST LONDON POST-GRADUATE COLLEGE, Prince of Wales's General Hospital, Tottenham, N.

**At 4.30 p.m.**

**Jan. 26,** Dr. G. N. Meachen, **The Dermatological Inspection of School Children.**

**Jan. 28,** Dr. T. R. Whipham, **Cases of Children's Diseases.**

### THE INFANTS HOSPITAL, Vincent Square, Westminster.

**At 3.30 p.m.**

**Jan. 26,** Dr. Vincent, **The Principles of Substitute Feeding as Applied to Healthy Infants.**

**Jan. 28 and 29,** Dr. Vincent, **The Methods of Dietetic Adjustment in, and Treatment of, Disorders of Nutrition in Infants.**

### LONDON SCHOOL OF CLINICAL MEDICINE, Seamen's Hospital, Greenwich, S.E.

**At 4 p.m.**

**Jan. 25,** Mr. Lawrence, **Hay Fever.**

**At 3.15 p.m.**

**Jan. 26,** Mr. Carless, **The Operative Treatment of Gall Stones.**

**At 2.15 p.m.**

**Jan. 29,** Dr. Rose Bradford.

THE HOSPITAL January 23, 1909.

*Name* ..................................................

*Address* ..................................................

..................................................

This Coupon must accompany manuscript or contributions intended for The Hospital.

